# Surface Emitting, Tunable, Mid-Infrared Laser with High Output Power and Stable Output Beam

**DOI:** 10.1038/s41598-018-36872-5

**Published:** 2019-01-24

**Authors:** Steven Slivken, Donghai Wu, Manijeh Razeghi

**Affiliations:** Center for Quantum Devices, Department of Electrical Engineering and Computer Science, Northwestern University, Evanston, IL 60208 USA

## Abstract

A reflective outcoupler is demonstrated which can allow for stable surface emission from a quantum cascade laser and has potential for cost-effective wafer-scale manufacturing. This outcoupler is integrated with an amplified, electrically tunable laser architecture to demonstrate high power surface emission at a wavelength near 4.9 μm. Single mode peak power up to 6.7 W is demonstrated with >6 W available over a 90 cm^−1^ (215 nm) spectral range. A high quality output beam is realized with a simple, single-layer, anti-reflective coating. The beam shape and profile are shown to be independent of wavelength.

## Introduction

Mid-wave infrared (MWIR) and long-wave infrared (LWIR) semiconductor lasers are being widely developed for chemical sensing and biomedical applications^1–5^. The main benefit of these devices is the ability to rapidly identify specific chemicals based on characteristic absorption features. The ideal laser for this type of application is compact, widely tunable, has a scalable power output, and is inexpensive to produce.

Monolithic edge-emitting quantum cascade laser (QCL) technology, which relies on cleaved facets for laser operation, has demonstrated wide tuning and high output power from a single aperture^6,7^. Sampled grating distributed feedback (SGDFB) technology^8,9^ provides non-mechanical tuning, and an integrated optical amplifier provides a high power option. However, testing and packaging of these devices require a labor intensive process which involves cleaving of individual laser cavities, mirror facet coating, and bonding of individual dies. As such, the economy of scale, though better than external cavity solutions, is still limited. A worthwhile goal is to develop wafer-scale fabrication to minimize die handling and greatly simplify pre-package screening, which in turn will minimize the cost to manufacture.

One method is to utilize a surface emitting laser architecture, which does not rely on wafer cleaving to operate. A commercially successful demonstration of this method in the near-infrared is the vertical cavity surface emitting laser (VCSEL), which can be produced in large quantities and 2-D arrays for high power illuminators^10^. Additional benefits of a surface-emitting laser include higher yield packaging and more uniform heat removal, as the edge quality is not critical and the device can be placed in the center, as opposed to the edge, of a heat spreader.

Unfortunately, due to polarization selection rules in the QCL, the traditional VCSEL architecture cannot be utilized. Grating-based outcouplers are another alternative, and have been demonstrated for the QCL^11^. Unfortunately, the device performance is very sensitive to the operating wavelength. Nevertheless, this architecture allows for the incorporation of laser and detector on the same chip and in the same beam path, which makes for a compact, easy-to-align, chemical sensor^12^. This is also highlighted in a recent publication, where a tunable laser and grating outcoupler were integrated to realize surface emission with monolithic beam steering^13^. In this case, the main focus was on the steering component, which was designed to eliminate massive optomechanical assemblies in directed energy applications. The grating outcoupler is not the preferred choice, however, if a stable output beam and tunable wavelength are both desired.

**Figure 1:**
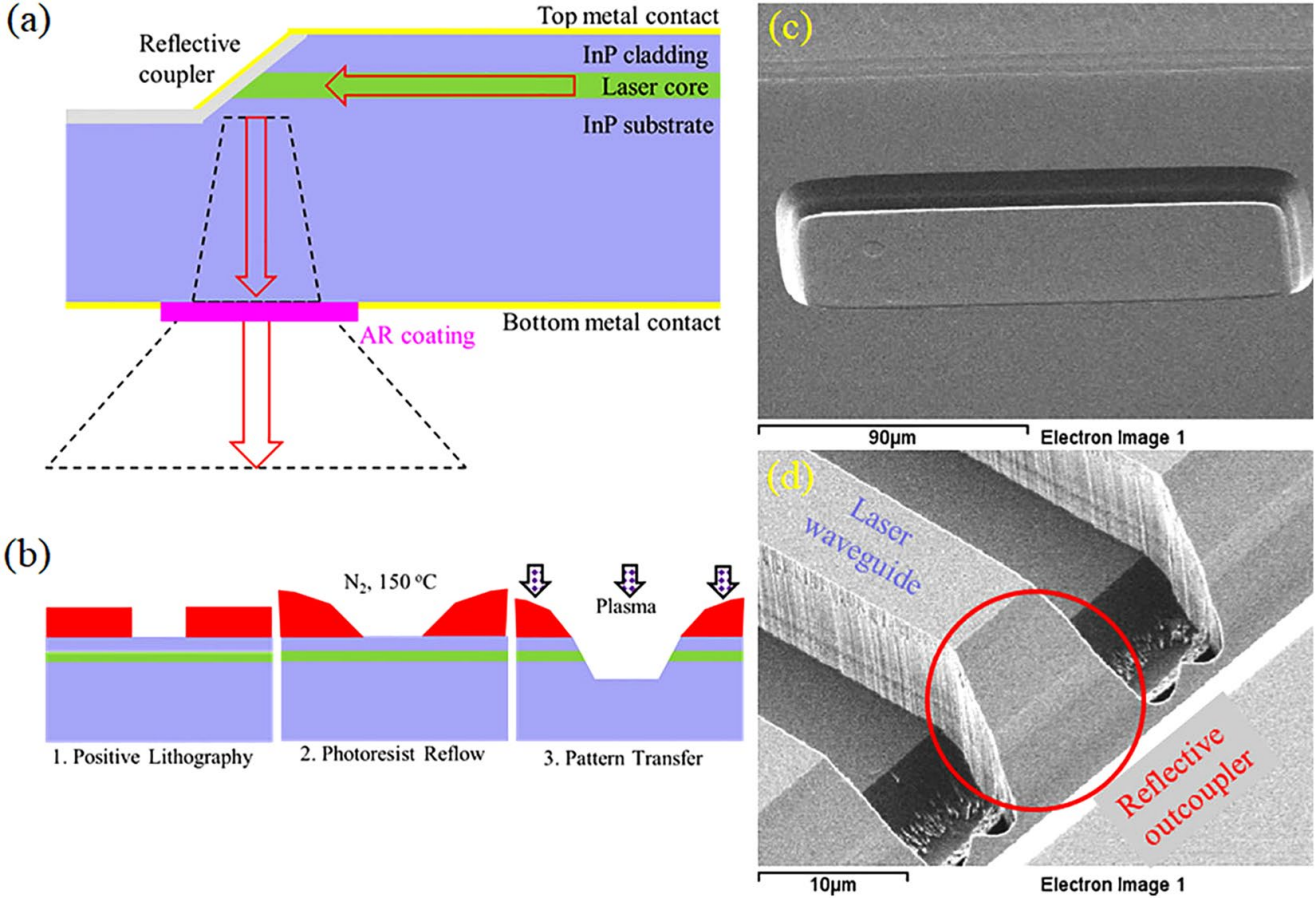
Reflective outcoupler operation and formation. (**a**) Schematic illustration of reflective outcoupler geometry. Red arrows indicate direction of light propagation. Dashed lines represent beam divergence in unguided regions. (**b**) 3 step illustration of mask shape transfer process. Oblique scanning electron microscope images of (**c**) a rectangular angled etch feature and (**d**) a reflective outcoupler formed at the intersection of etched features.

An alternative strategy is to utilize a reflective outcoupler, which takes in-plane light and redirects it either out of the top surface or through the laser substrate. (see Fig. 1a) This approach has been used for near-infrared lasers in the past, including the in-plane surface emitting laser (IPSEL), folded cavity surface emitting laser (FCSEL), and horizontal cavity surface emitting laser (HCSEL)^14–16^. However, the methods used have the drawback of process complexity and are not very well suited for large-scale wafer processing. A chemical etching technique was also recently explored for producing the reflective outcoupler in a InP/InGaAsP laser^17^, but the process is crystal facet-limited to produce a 55° angle, which limits the versatility of the technique. In addition, this approach is not easily adapted to the QCL waveguide, which has a more complex waveguide containing InP, GaInAs, and AlInAs.

An alternative technique, explored in this work, is to use mask shape transfer to create reflective outcouplers in the semiconductor waveguide^18^. This technique has been used for microlens manufacture previously, is compatible with a standard plasma etching system (for large area manufacturing), and can generate a wide variety of facet angles. As proof-of-concept, this outcoupler is integrated with a tunable QCL and an optical amplifier to demonstrate high peak power (>6 W), stable surface emission in the MWIR.

## Results

### Reflective outcoupler formation

The facet fabrication technique is shown schematically in Fig. 1b. A lens shape is formed in the photoresist mask by thermal reflow (See Methods). Plasma etching is then used to transfer the photoresist shape into the semiconductor crystal with a controlled selectivity. In addition to controlling the shape of the etch sidewall, the photoresist reflow technique also helps remove mask micro-roughness, which creates a smoother edge profile.

An example of an etched rectangular feature is shown in Fig. 1c. Smooth sidewalls are demonstrated, with no preference for crystal orientation. Cross-section analysis of the etched feature used gives a laser core angle of ~53 degrees with respect to the wafer surface. The outcoupler angle was reproduced within measurement accuracy (~1 degree) in two separate fabrication runs. Uniformity of etch angle is found to be limited only by the uniformity of the photoresist thickness, as long as thermal contact is uniform across the wafer during plasma etching.

Once the angled feature is formed, a double channel laser waveguide can then be defined with a separate plasma etching process. This process forms a vertical-walled waveguide which intersects with the angled sidewall, forming a reflective outcoupler termination, as shown in Fig. 1d. No significant roughness (low rugosity) is observed on the outcoupler feature, which is significantly smoother than the vertical waveguide sidewall.

**Figure 2:**
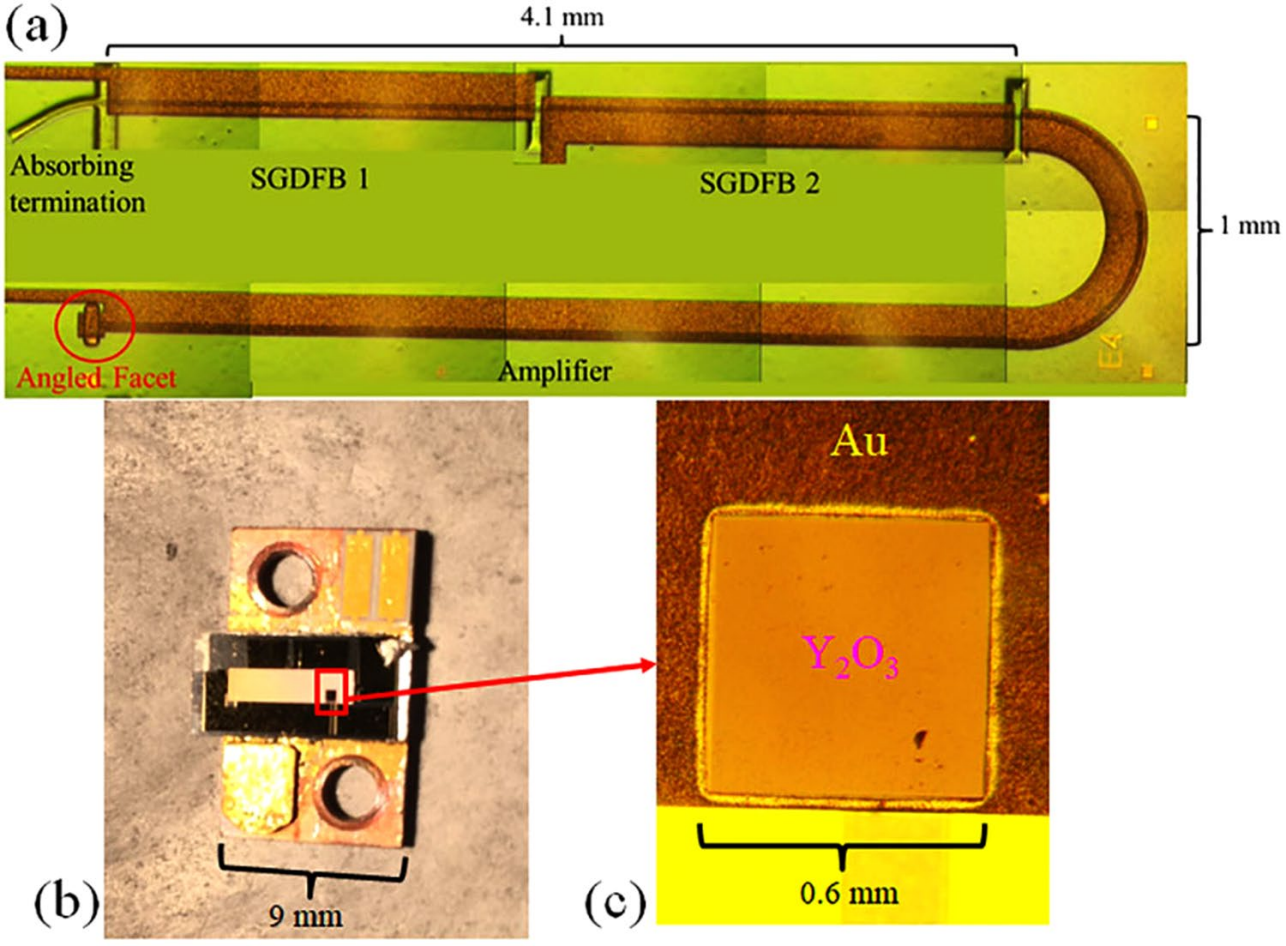
Images of laser after fabrication and packaging. (**a**) Composite top view microscope image of the amplified, tunable laser. (**b**) Image of an epilayer-down packaged, substrate-emitting laser. (**c**) Magnified image of an AR-coated substrate emission window.

The wafer was fabricated into an amplified, tunable laser architecture which is illustrated in Fig. 2a. The device is composed of three active sections, an absorbing termination, and the reflective outcoupler. The SGDFB section and absorbing termination design and fabrication are identical to that used in ref.^13^, with the exception that the grating period used for this device was 775 nm. The amplifier section is composed of a curved semicircular (r = 500 μm) waveguide with a width of 5 μm, a waveguide width taper from 5 to 10 μm over a distance of 50 μm, and a straight waveguide with a 10 μm width and 4.1 mm length. The reflective outcoupler feature was formed (to a depth of 10 μm) by means of mask shape transfer prior to waveguide definition, as described in the Methods section. The final fabrication steps follow the procedure established in ref.^13^ and are also summarized in the Methods section. A single layer of Y_2_O_3_ was used as an anti-reflection (AR) coating for packaged devices.

**Figure 3:**
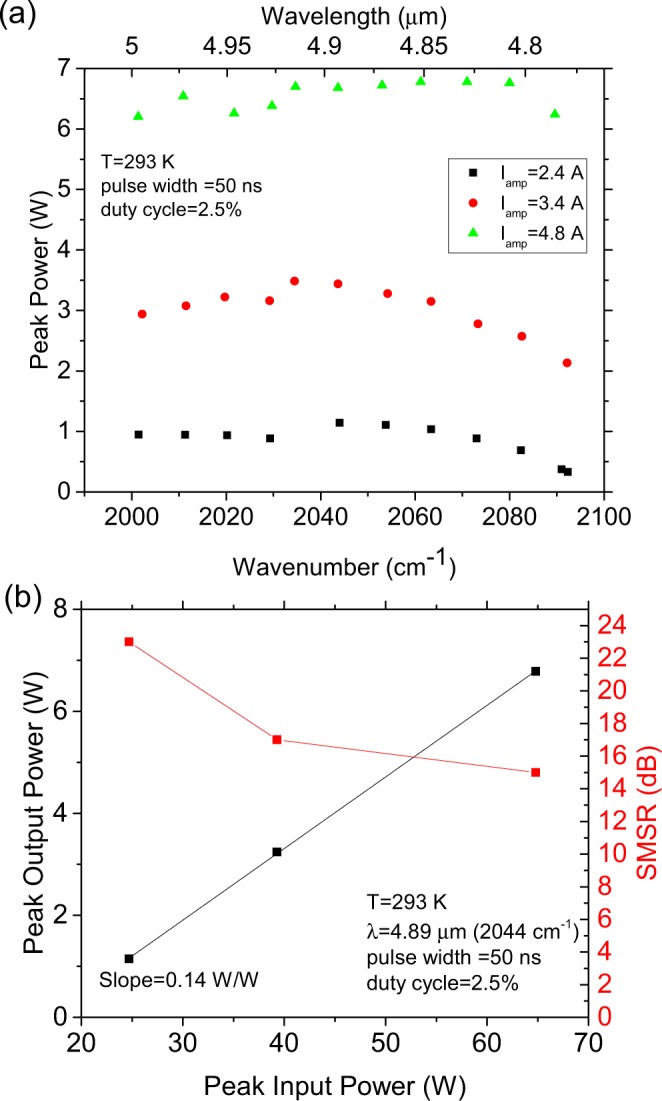
Tuning and power amplification characteristics of the surface emitting laser. (**a**) Peak output power as a function of wavenumber (wavelength) for different values of the amplifier current. (**b**) Peak output power and SMSR as a function of amplifier input power at λ = 4.89 μm.

Similar to the demonstration in ref.^13^, the SGDFB design is capable of single mode emission and electrical tuning over a 90 cm^−1^ (215 nm) spectral range. At low amplifier current, the side mode suppression ratio (SMSR) is above 20 dB over most of the tuning range. The output power increases as a function of amplifier current is shown in Fig. 3a. Single mode peak power up to 6.7 W is demonstrated with >6 W available over the entire tuning range at an amplifier current of 4.8 A. An SGDFB device without an integrated amplifier from the same process exhibits an output power of only 0.1–0.2 W over the tuning range. The peak output power and SMSR as a function of peak amplifier input power are shown in Fig. 3b for λ = 4.89 μm. Output power increases linearly over the tested range. From the slope of the output power data, it is observed that the amplifier power conversion efficiency is 14%. Some degradation of the SMSR is observed at higher currents, but it remains above 15 dB at the highest power output. This initial demonstration of the reflective outcoupler was made in pulsed operation using a wafer that was optimized for high peak power operation (see Methods). However, the device waveguide and packaging are very similar to previously demonstrated devices which achieved continuous wave (CW) operation^7^. CW operation is feasible for this architecture as well with an optimized wafer design.

The far field for a laser with a reflective outcoupler is ideally the same as a cleaved device with the same output waveguide. As the outcoupling is due to total internal reflection, the beam direction should also not change with output wavelength. Distortions of the beam shape that may occur are from distortion/scattering in the reflective outcoupler and interference at the air/substrate interface.

**Figure 4:**
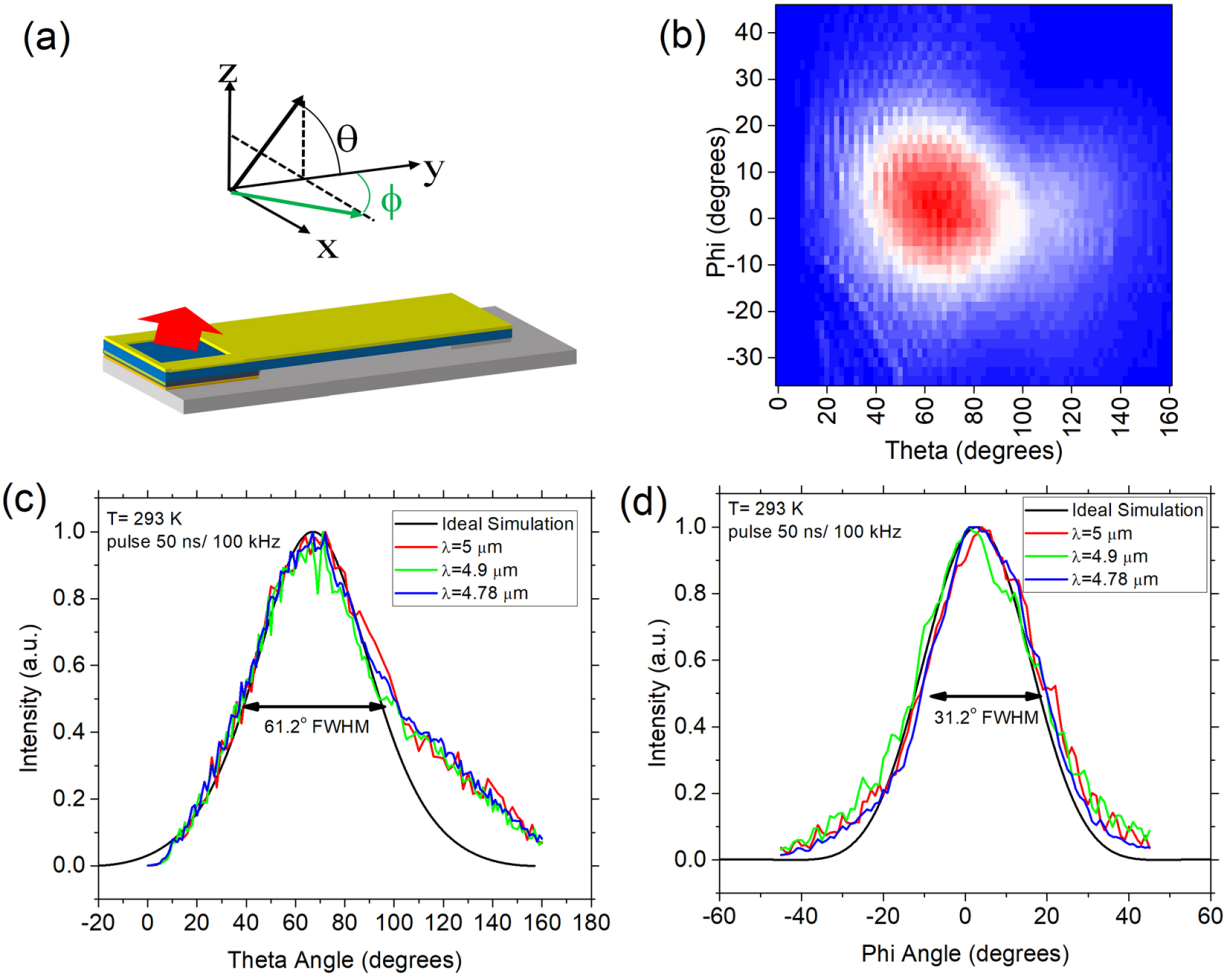
Far field characteristics of the surface emitting laser. (**a**) Far Field measurement geometry for the surface emitting laser. (**b**) Measured 2D far field pattern. 1D far field scans are shown in (**c**) phi and (**d**) theta directions at different wavelengths and compared with ideal edge emitter simulations. All curves are measured at an amplifier current of 4.8 A.

For devices without AR coating on the substrate, significant interference effects were observed. AR-coated lasers, on the other hand, show fairly smooth profiles. A typical 2-dimensional (2D) far field of the surface emitting laser, measured at an amplifier current of 4.8 A, is shown in Fig. 4b. The intensity is highest at a theta angle of 67 degrees (peak angle), which matches well to the expected peak angle (64 degrees) based on the measured outcoupler angle of 53 degrees. The far field was measured for multiple devices from different sections of the processed piece, and all had the same peak angle. The high angle tail in the theta direction is due to a small discontinuity in etch rate between the laser core and cladding material. This creates some smearing of the beam, which can be remedied with further process development.

The full width at half maximum (FWHM) of the output beam is 31.2 and 61.2 degrees in the phi and theta directions, respectively. Measurements of the far field at lower power are almost identical. Beam divergence values are very similar to the simulated ideal beam divergence from an edge emitting QCL with the same waveguide (30.5 and 54 degrees, respectively). This is a relative change of 2.3% (13.3%) in the phi (theta) direction. A graphical comparison is shown in Fig. 4.

Besides being stable with output power, the far field is also stable with respect to output wavelength. Experimental 1D far field curves at several wavelengths are overlaid in Fig. 4 and compared with the ideal simulated far field from a cleaved waveguide. All curves are measured at an amplifier current of 4.8 A. The simulated curve is offset in angle for better comparison.

## Discussion

While the device architecture does not allow for a straightforward direct comparison to an edge emitting laser, we can estimate the outcoupling efficiency of the reflective outcoupler. The main losses that arise with the reflective outcoupler geometry are scattering at the outcoupler and free carrier absorption in the substrate. Due to the extremely smooth outcoupler sidewall, which is shown in Fig. 1d, the actual scattering loss is not expected to be significant. If present, it would lead to a large diffusive and/or speckled background in the far field, which is not observed

On the other hand, the free carrier losses in the substrate do play a role in the power outcoupling efficiency. The substrate doping (1.4 × 10^17^ cm^−3^) leads to an estimated free carrier absorption of 6.6 cm^−1^ at λ = 4.9 μm. For a 200 μm thick substrate, this leads to an outcoupling efficiency of 87.6%. This thickness was chosen simply to minimize the chance of breakage during fabrication. Reducing the substrate thickness to 100 μm would increase the outcoupling efficiency to 93.6%. Another possibility is to switch to a semi-insulating substrate, which would require another top contact, but reduce the losses. A third option is to incorporate a partial via above the outcoupler in order to extract the light closer to the waveguide.

Beyond this demonstration, the mask transfer technology can also be developed for other applications. One aspect to be explored is the fact that the reflector angle is adjustable, and can be shaped dynamically during etching. Besides straight facets, there is an opportunity to create a more parabolic profile with a single etch process, which can be used to increase or decrease the beam divergence at the output of the waveguide^19^. Another opportunity that can be explored is the manufacture of refractive lenses directly on the wafer substrate^20^. In this case, the lens would occupy the same area as the substrate emission window, with a radius of curvature adjusted to directly collimate the laser output.

In conclusion, a cost-effective technology for wavelength-independent surface emission from a quantum cascade laser has been developed. A reflective outcoupler is fabricated via a mask transfer process in a standard plasma etching system. This outcoupler is integrated with an amplified, electrically tunable laser architecture to demonstrate high power surface emission at a wavelength near 4.9 μm. Single mode peak power up to 6.7 W is demonstrated with >6 W available over a 90 cm^−1^ (215 nm) spectral range. A high quality beam is also demonstrated, with a beam divergence within 14% of the diffraction limit.

## Methods

### Device fabrication

A high efficiency MWIR (λ ~ 4.9 μm) QCL wafer is employed, similar to that used in ref.^13^, which includes an integrated grating layer above the waveguide core. The main difference is the doping in the laser core, which was ~2 times higher in order to target high peak power operation.

Device fabrication starts with diffraction grating formation for the SGDFB laser sections using e-beam lithography and plasma etching. The SGDFB section and absorbing termination design and fabrication are identical to that used in ref.^13^, with the exception that the grating period used for this device was 775 nm. The sampling periods used for section 1 and 2 were 170.9 and 158.8 μm, respectively. After patterning, the top InP waveguide cladding and cap layers were grown using low pressure metalorganic chemical vapour deposition, with same thickness and doping used in ref.^13^.

Reflective outcoupler fabrication follows the illustration of mask shape transfer given in Fig. 1b. A thick, positive photoresist (AZ 10XT) is patterned by photolithography. The photoresist is reflowed in a tube furnace under nitrogen ambient at 150 °C to create a lensed shape with a variable angle near the base. A BCl_3_:Ar plasma that can etch both photoresist and semiconductor is then used to transfer the mask shape into the semiconductor. The outcoupler shape can be magnified or demagnified in the vertical direction by controlling the selectivity ratio (ratio of etch rates between the semiconductor and mask) of the plasma. The selectivity can be adjusted through flow ratio, system pressure, applied power, and temperature. This allows the same mask to produce semiconductor facets with variable angles. Angled sidewall features were etched in a PlasmaTherm SLR-770 ECR-RIE plasma etching system.

Electrical isolation regions and double channel waveguide formation for the SGDFB laser, absorbing termination, and amplifier followed the same procedure as in ref.^13^ after careful alignment to the reflective outcoupler using photolithography. A standard insulation and top contact metallization scheme were employed, as described in ref.^13^. Following this, the substrate was thinned to 200 μm and polished. Bottom contact formation included the definition of emission windows aligned to the reflective outcouplers in order to allow for direct substrate emission. The bottom contact metal consists of a multilayer of AuGe/Ni/Au.

### Device Testing

The resistances of all devices were checked prior to die singulation and packaging, which led to excellent testing yield and improved efficiency. Dies were bonded epilayer-down to patterned diamond heat spreaders with indium solder, as shown in Fig. 2b. This provides easy access to substrate emission and excellent heat removal. After packaging, a 690 nm Y_2_O_3_ layer was deposited on the substrate via ion beam deposition. This serves as an anti-reflective coating for substrate emission, which removes most of the interference effects from the output beam far field.

Testing was performed in pulsed mode (50 ns, 2.5% duty cycle), with an independent, isolated pulse driver for each active laser section. The emission wavelength is electrically tuned by adding a controlled, independent dc bias to one or both SGDFB sections. The average power output from the substrate was measured at different wavelengths and amplifier currents using a calibrated thermopile. No correction for power collection was made, and peak power was calculated based on the pulse duty cycle (2.5%). SGDFB laser output wavelength was measured using a vacuum FTIR (Bruker IFS 66 v/S) with a HgCdTe photodetector and a maximum resolution of 0.125 cm^−1^ to ensure single mode operation for steering measurements. Far fields patterns were obtained with a computer-controlled dual axis goniometer and a InSb photovoltaic detector placed 230 mm from the device with a 1.3 mm square input aperture.

## Data Availability

The datasets generated during and/or analysed during the current study are available from the corresponding author on reasonable request.
